# Morphological and Genetic Diversity of Cucumber (*Cucumis sativus* L.) Fruit Development

**DOI:** 10.3390/plants12010023

**Published:** 2022-12-21

**Authors:** Rebecca Grumet, Ying-Chen Lin, Stephanie Rett-Cadman, Ajaz Malik

**Affiliations:** 1 Graduate Program in Plant Breeding, Genetics and Biotechnology, Department of Horticulture, Michigan State University, East Lansing, MI 48824, USA; 2 Department of Horticulture-Vegetable Science, Sher-e-Kashmir University of Agricultural Sciences and Technology of Kashmir, Shalimar, Srinagar 190 025, India

**Keywords:** cucumber, *Cucumis*, fruit development, ovary development, fruit shape, spines, cuticle

## Abstract

Cucumber (*Cucumis sativus* L.) fruits, which are eaten at an immature stage of development, can vary extensively in morphological features such as size, shape, waxiness, spines, warts, and flesh thickness. Different types of cucumbers that vary in these morphological traits are preferred throughout the world. Numerous studies in recent years have added greatly to our understanding of cucumber fruit development and have identified a variety of genetic factors leading to extensive diversity. Candidate genes influencing floral organ establishment, cell division and cell cycle regulation, hormone biosynthesis and response, sugar transport, trichome development, and cutin, wax, and pigment biosynthesis have all been identified as factors influencing cucumber fruit morphology. The identified genes demonstrate complex interplay between structural genes, transcription factors, and hormone signaling. Identification of genetic factors controlling these traits will facilitate breeding for desired characteristics to increase productivity, improve shipping, handling, and storage traits, and enhance consumer-desired qualities. The following review examines our current understanding of developmental and genetic factors driving diversity of cucumber fruit morphology.

## 1. Introduction

Cucumber (*Cucumis sativus* L.) plants are cultivated throughout the world for immature fruit that are eaten fresh or processed into pickles. The fruit, which are formed from an inferior ovary, are generally harvested toward the end of the period of exponential growth, approximately two weeks post-floral anthesis. Flesh tissue of the fruit at this stage is crisp, and seeds remain small and tender, allowing for consumption of the full fruit. Unlike fruits that are eaten ripe, where metabolic attributes such as sweetness, flavor, and aroma are key quality determinants, the predominant characteristics that drive cucumber fruit quality are morphological: size and shape; external features, such as waxiness, spines and warts; and internal features, such as flesh thickness and seed cavity size. 

**Figure 1:**
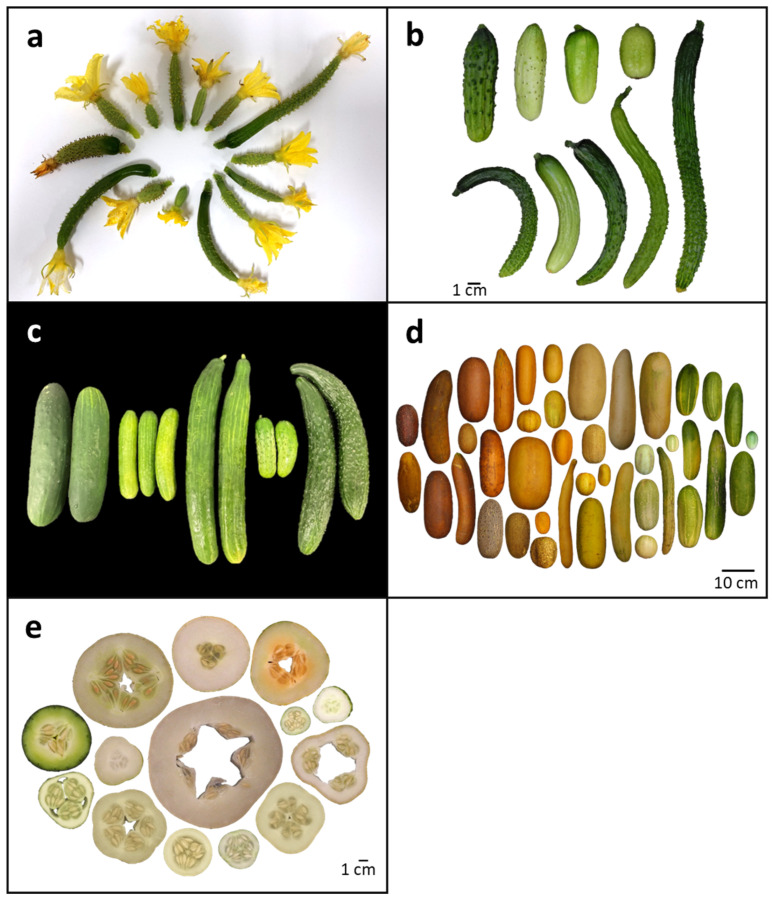
Examples of diversity in cucumber ovary and fruit morphology at different stages of development as observed in the CucCAP cucumber core collection [1]. (**a**) Cucumber ovaries at anthesis or one day post-anthesis exhibit differences in size, shape, presence/absence/number of spines, spine color, warts, and ribs. (**b**) Variation in cucumber fruit development during early exponential growth 5–8 days post pollination (dpp). (**c**) Examples of cucumber market types. Left to right: Western fresh market (slicing); Beit Alpha/Mediterranean; parthenocarpic greenhouse; Western pickling; Chinese Long. (**d**) Mature cucumber fruit vary in shape, size, color, surface texture, and netting. (**e**) Variation in internal properties at maturity (flesh thickness, seed cavity size, color, and hollows).

These morphological features are evident in the different types of cucumbers that are preferred throughout the world (Figure 1). East Asian cucumbers, which are eaten fresh, typically have very long fruits, such as the North China type, Chinese Long. North American cucumbers for fresh markets (also called slicing cucumbers) are characterized by smooth, intermediate length fruit (20–30 cm), while North American and European pickling cucumbers have warty, short fruit (5–15 cm). Parthenocarpic, greenhouse-grown cucumbers include two additional fresh market classes, long-fruited (30–40 cm) cucumbers and Beit Alpha or Mediterranean cucumbers, with short (12–15 cm) fruit. Both parthenocarpic types are thin-skinned and typically require protective covering to reduce water loss during shipping and handling. In addition to the predominant market classes, there is extensive diversity in fruit morphology within the cucumber germplasm (Figure 1). However, consumer preferences and handling requirements for packers and shippers prioritize uniform, straight fruit that are readily sorted and processed within the market chain, limiting the diversity of fruit type that is commercially produced. 

The past decade has been marked by rapidly increasing genomic and biotechnological tools for cucumber and other cucurbit crops including genome assembly and annotation, extensive transcriptomic analyses, and development of multifunctional genomic databases allowing for genome-wide analyses of gene expression, identification of genetic variants, and examination of cross-species synteny. This has resulted in tremendous advances in our ability to map genomic regions, monitor gene expression, and postulate and test candidate genes for important traits. The following review will examine our current understanding of developmental and genetic factors driving diversity of cucumber fruit morphology.

## 2. Fruit Development, Size, and Shape

**Figure 2:**
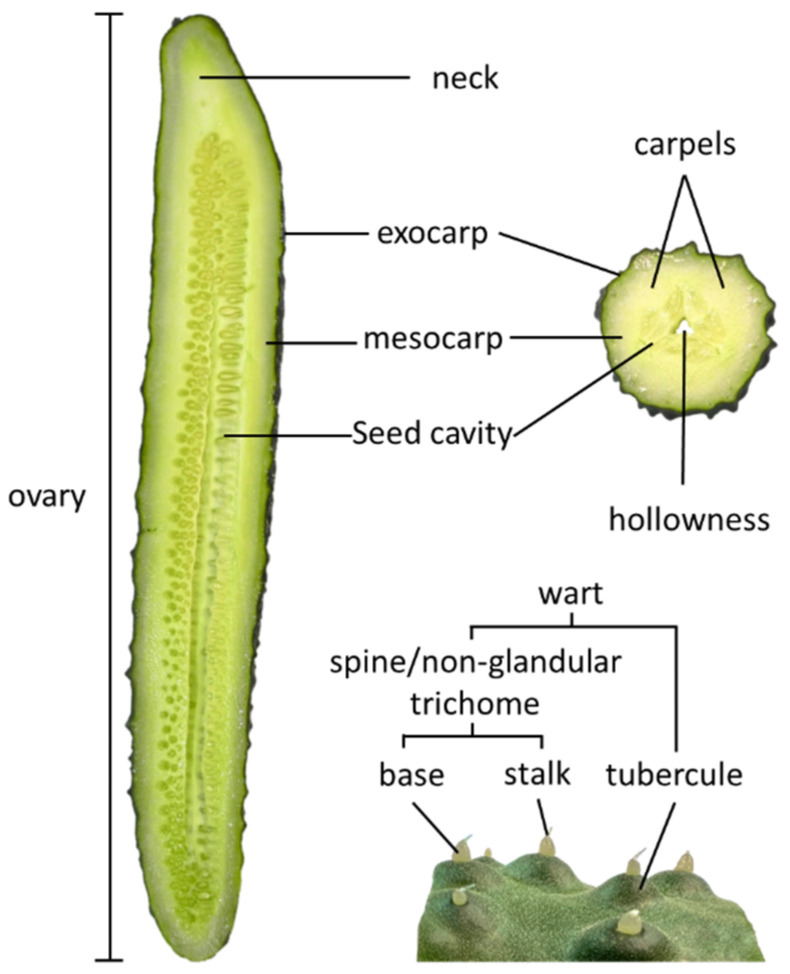
Structural features of cucumber fruit in longitudinal and cross-section.

Cucumber fruits and other cucurbit family fruits are characterized as pepo, having a fleshy, many-seeded interior and a hard or firm rind [2]. The fruit, or pericarp, is composed of exocarp (outer layer, i.e., skin or rind); mesocarp (flesh); and endocarp surrounding the ovules or seeds (Figure 2). Cucumber ovaries, which carry the seeds, are typically comprised of three fused carpels. Superimposed on this basic structure, numerous factors during floral and fruit development confer the observed diversity of fruit size and shape.

### 2.1. Fruit Size

Organ size results from a combination of both cell number and cell size. For cucumber fruits, cell number is primarily established during ovary development, with a second phase of cell division during the first 4–5 days post pollination [3,4,5]. A 7–80-fold increase in cell number in the longitudinal axis was observed in cucumber ovaries from seven days pre-anthesis to anthesis; a further 2–3-fold increase occurred during the first four days post-pollination [4,5]. The number and orientation of cell divisions that occur during ovary development provide the foundation for subsequent fruit size and shape, and genetically determined differences are readily apparent in the developing ovary at the time of floral anthesis (Figure 1a). Co-localization of quantitative trait loci (QTL) for cucumber ovary length and diameter with mature fruit length and diameter support the shared genetic basis for size and shape of ovaries and fruits (e.g., [6,7,8]). 

Differential expression of genetic factors influencing cell division, such as cell cycle genes and genes associated with chromosomal alignment at mitosis, have been associated with differences in cucumber fruit size [9,10,11]. Increased expression of four cyclin genes and enrichment for microtubule-based movement-associated genes were observed in near-isogenic, long- vs. short-fruited cucumber lines [3]. Variation in expression of mitotic kinesins was observed in cucumber ovaries in correlation with differences in cell division [12]. A series of short fruit (*sf*) mutants also have been found to result from reduced cell number. Map-based cloning of *sf* mutant 3 (*sf3*) identified an amino acid substitution in a katanin p60 subunit (CsKTN1) [13], a factor that interacts with microtubules and has been implicated to influence a range of cell functions including spindle formation during mitosis [14]. The *sf2* phenotype results from a single nucleotide mutation in histone deacetylase complex 1 protein (HDC1) that is predominantly expressed in meristematic tissue and influences expression of genes associated with cell cycle regulation [15]. Similarly, an alternate allele of the FRUITFUL-like MADS-box gene (*CsFUL1^A^*), which was associated with longer ovaries and fruit length in East Asian cucumbers, has been shown to bind to the promoter of *SUPERMAN*, a regulator of cell division and expansion [16].

**Figure 3:**
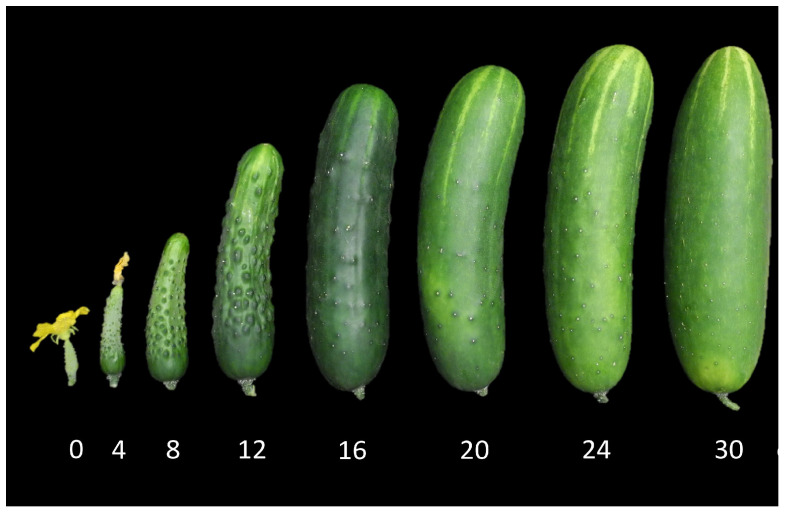
Growth of cucumber (cv. Poinsett) fruit from 0–30 days post-pollination (dpp).

Subsequent to cell division, cucumber fruits enter a 1–2 week period of exponential growth marked by rapid expansion in cell and fruit size; full length is generally reached approximately two weeks post-pollination (Figure 1b and Figure 3) [4,5,17]. Water and photosynthate are actively transported into the developing sink tissue. Key genes influencing sugar transport into the fruit, e.g., *CsSWEET7a* mediating apoplastic phloem unloading, and alkaline alpha-galactosidase 2 (*CsAGA2*), which hydrolyzes sucrose into glucose and fructose, are highly expressed in growing cucumber fruits [18,19]. Down-regulation of these genes by RNAi caused a delay in fruit growth, while overexpression increased growth. Modulating expression of *AGA2* also influenced photosynthetic rate of source leaves, indicating importance of sink–source communication. The rate of transport of sugars also has been influenced by domestication [19]. Cultivated cucumbers are associated with higher levels of glucose and fructose than wild; and genomic analyses indicate a selective sweep causing increased expression of the *AGA2* gene in cultivated cucumbers.

Competition for photosynthetic resources also influences fruit growth. First fruit inhibition is a long-recognized phenomenon in cucumber, wherein initial fruit set, either pollinated or parthenocarpic, can inhibit subsequent fruit set or cause a significantly reduced growth rate [20,21,22]. Sink activity, including activity of AGA2, accumulation of sucrose, and accumulation of the source–sink signal molecule, trehalose-6-phosphate (T6P), was found to be greater in first fruit than second fruit [20]. Extensive metabolic and transcriptional changes were observed within 24 h post-pollination but differed markedly between the first and second fruit. Removal of the first fruit released inhibition of the second fruit, resulting in increased growth rate, sink activity, and a reprogramming of the metabolome to resemble early post-pollination in a non-inhibited fruit [20,21].

Not surprisingly, several plant hormones which can influence both cell division and cell expansion have long been recognized to play important roles in stimulating or inhibiting cucumber fruit growth (e.g., [5,10,17,22]). Auxins, gibberellins, cytokinins, and brassinosteroids are more frequently associated with fruit growth, while ethylene is associated with growth inhibition. Auxin (IAA) content was positively correlated with fruit length in a set of cucumbers with diverse fruit size and shape [5], and inhibition of expression of auxin transporters and decreased auxin levels in the fruit were observed in the short fruit of *CsFUL1^A^* [16]. Numerous studies also show interconnection among hormones and other factors influencing fruit growth. The microtubule-associated short fruit phenotype of *sf3* was associated with modified expression of auxin and gibberellin-related genes [13], and the *sf2* mutant showed histone deacetylation associated with key genes involved in cytokinin and polyamine pathways [15]. Mutation of the *sf1* gene, which encodes a RING-type E3 ligase, resulted in accumulation of the ethylene biosynthetic enzyme ACS2 and overproduction of ethylene [23]. Similarly, second fruit inhibition was associated with transcripts involved in gibberellin degradation and cytokinin conjugation consistent with inhibition of growth, while in first fruit, inhibitory ethylene transcription factors were down-regulated [21]. 

Recent studies also implicate potential involvement of numerous micro(mi)RNAs in regulation of cucumber fruit expansion [24,25]. Targets of miRNAs differentially expressed during rapid fruit growth included numerous transcription factors associated with fruit development in several species [25]. Some examples, e.g., *csa-miR156a*, which targets SPL/SBP-box transcription factors; *csa-miR160*, which targets auxin response factor 7; and *csa-MIR319b*, which targets MYB and TCP transcription factors, were down-regulated in expanding cucumber fruit. Others, such as *miR164* and *miR166*, which target NAC domain transcription factors and an ABC transporter C family member, respectively, showed higher expression in expanding cucumber fruit, presumably relieving inhibitory effects of these factors.

The complexity of the fruit growth process and potential involvement of a wide number of genes involved in cell division, cell expansion, hormone signaling, and accumulation of photoassimilates is further supported by the large number of QTL that have been identified in studies of cucumber fruit size. Numerous investigations have found as many as 10–20 fruit size QTL, and several hot spots for consensus fruit size loci have been identified [6,8,26,27,28,29,30]. In many cases these QTL also cluster with QTL for specific anatomical fruit traits such as peduncle length, neck length, seed cavity size, and flesh thickness, all of which can influence fruit size. Extensive reviews of the literature [28,30] collated 135 QTL identified for fruit size (fruit length, fruit diameter, or fruit size) in cucumber along with their chromosomal locations (cucumber genome Gy14v.2). Nineteen consensus QTL spanning all seven chromosomes were identified. Nine of the QTL were detected throughout fruit development, ovaries, immature, and mature fruit. Each of the consensus QTL included orthologs of known meristematic and fruit size and shape genes identified in other species, such as *SUN* (encoding an IQ67 calmodulin-binding domain that influences the direction of cell division), *WOX* (WUSCHEL-related homeobox proteins that regulate meristem size), *CLV3* (CLAVATA3, peptide signal regulating meristem activity), *YABBY* (transcription factor family associated with abaxial/adaxial polarity and boundary formation), *CRABSCLAW* (carpel development regulator), and *TRM* (TONNEAU1 Recruiting Motif protein), that interact with OVATE to modulate cell division by sub-cellular compartmentalization. However, in most of these cases, multiple candidate genes occur within a QTL region and the specific responsible genes remain to be identified. Clustering of size- and shape-related genes within a small chromosomal region also has been observed in tomato [31,32], and syntenic blocks of gene clusters have been identified across cucurbit species including cucumber, melon, and watermelon [6,28,30].

In addition to the examples above, genes that more broadly affect cucumber plant morphology can also influence fruit size, as illustrated by a few recently characterized systems. CLAVATA1 (CLV1) is a shoot apical meristem protein that influences stem cell fate, in turn, modulating features such as phyllotaxy, branching pattern, organ initiation, and shoot determinancy [33]. An EMS-induced cucumber mutation of *CsCLV1* was found to cause a variety of morphological changes including abnormal phyllotaxy, fascinated stems, increased number of floral organs, and shorter and thicker fruit [34]. The *littleleaf* (*ll*) mutant of cucumber causes smaller leaves and other organs and increased lateral branching [35]. The smaller organs, including smaller fruit, result from a reduction in cell number and cell size. *LL* was found to encode an ortholog of Arabidopsis *STERILE APETALA*, a WD40 repeat domain containing protein that functions in organ size control [35]. In another example, knockdown of a basic Helix-Loop-Helix (bHLH) protein, Irregular Vasculature Patterning (CsIVP), resulted in irregular vascular patterning and abnormal organ development, including dwarf plants, curled leaves, reduced ovary and fruit length, and decreased seed viability [36].

### 2.2. Parthenocarpy

Parthenocarpy is a naturally occurring phenomenon in some plants where development of fruit occurs without fertilization. Parthenocarpy can be obligatory or facultative, the latter being present when pollination is absent or has been prevented [37]. This trait is critical under conditions that are inappropriate or hostile to pollinators, including greenhouse cultivation that restricts entry of external biota. Parthenocarpic cucumber varieties such as the European greenhouse and Beit Alpha types are widely used for greenhouse production of cucumbers. Parthenocarpy can also facilitate early- or late-season production, increasing availability to consumers and revenue to farmers [38].

Numerous studies from the mid-1900s demonstrated that parthenocarpic fruit set in cucumber can be induced with the application of various exogenous growth-promoting phytohormones including auxins, cytokinins, gibberellic acid, and brassinosteroids, and that combinations of hormones can increase success. Accordingly, increased transcription of genes involved in auxin, GA, and cytokinin biosynthesis has been observed in genetically parthenocarpic fruit as well as hormone-induced parthenocarpy [39,40]. Auxins appear to have a central role, as concentration of auxins and expression of auxin-producing proteins were greater in parthenocarpic fruit and treatment with auxin resulted in elevated levels of gibberellins and cytokinins in parthenocarpically-induced fruit [39,40]. Consistent with early observations showing that inhibitors of auxin transport that block movement of auxin out of the ovary can promote parthenocarpy [41], parthenocarpic cucumbers exhibited decreased transcription of the auxin receptor and signal transport genes *CsTIR1* and *CsAFB2* [42]. Interestingly, hormone-insensitive parthenocarpy also has been observed in certain lines of cucumber, possibly resulting from expression of proteins that showed hormone-insensitive patterns in both ovaries and seedlings [43].

As is the case for fruit set following pollination, import of sugars is critical for parthenocarpic fruit growth. Increased sugar transport was associated with increased expression of auxin and cytokinin signaling genes suggesting interplay among sugars and plant hormones [44], and a higher rate of cell division in parthenocarpic fruit was associated with higher carbohydrate metabolism [43]. In some genetically parthenocarpic lines, especially European greenhouse types, parthenocarpy has been associated with weaker first fruit inhibition (e.g., [45]), potentially providing a yield advantage. 

Fortunately, there exists a rich parthenocarpic germplasm in cucumber facilitating development of parthenocarpic varieties [40,46]. A screen of diverse germplasm found a range in frequency of parthenocarpic fruit set from less than 25% to nearly 100% [45]. Genetic studies indicate complex inheritance with multiple QTL, and variable results have been obtained depending on environmental conditions and germplasm studied. Sun et al. [47] identified ten QTL associated with parthenocarpy distributed across four genomic regions and Wu et al. [48] reported seven QTL for parthenocarpic fruit set with a major-effect QTL, parth2-1 on chromosome 2; Lietzow et al. [37] identified four QTL consistently linked with parthenocarpic fruit set in North American processing cucumber which showed incomplete overlap with the QTL detected in the European greenhouse genotypes; and Niu et al. [49] reported four new parthenocarpy QTL from a South China ecotype cucumber. Six loci were identified in a genome-wide association study (GWAS) of 31 cucumber lines [45]. Candidate genes regulating parthenocarpy have not yet been identified. 

### 2.3. Fruit Shape

Commercially produced cucumbers are typically elongated and cylindrical; however, cucumber fruit can vary extensively in several factors influencing shape including length, diameter, uniformity (i.e., cylindrical vs. tapered), and tendency to curve (Figure 1). Consistent with the relationship between ovary and fruit shape, organ features such as floral sex and carpel number that are established during floral meristem development can directly influence cucumber fruit shape [50]. Cucumber plants are typically monoecious, producing separate male and female flowers on the same plant. Female flowers result from suppression of the anther whorl by the *M* (*Monoecious*) locus during floral primordia development. *M* encodes an ethylene biosynthetic enzyme, ACS (1-aminocyclopropoane-1-carboxylate synthase; CsACS2), whose expression is localized to the developing ovary primordia of carpel-bearing flowers [51,52,53]. The occurrence of bisexual flowers, either in hermaphrodite plants or andromonecious plants which produce separate male and bisexual flowers, results from the presence of the recessive *m* allele encoding an enzyme with reduced biosynthetic activity [52]. Fruit produced from bisexual flowers are typically rounder than those from female flowers, a phenomenon that is also observed in other cucurbit crops including melon (*Cucumis melo*) and squash (*Cucurbita pepo*) [28,54,55]. However, there are also examples of hermaphrodite cucumber mutants and transgenic melons with bisexual flowers with elongated fruit [56,57], suggesting that fruit shape does not directly result from accommodation of the stamen whorl during fruit development but may involve a complex interplay between timing and expression of ethylene production and ovary development. 

While ovaries of cucumber flowers are generally comprised of three fused carpels, mutation of the semi-dominant carpel number (*CN*) gene results in five carpels and produces a rounder fruit [58]. *CN* has been identified to be an ortholog of *WUSCHEL*, a regulator of meristem size in Arabidopsis [58,59]. The *CLAVATA3* and *FRUITFUL1* genes, *CsCLV3 and CsFUL1(A)*, were also shown to influence carpel number in cucumber [60]. Increased carpel number resulted from decreased expression of *CsCLV3* or overexpression of *CsFUL1(A)*. Biochemical analysis showed interactions between all three genes leading to regulation of carpel number. CsFUL1(A) binds to the promoter of *CsWUS* to stimulate expression; CsWUS, in turn, binds to the promoter and activates expression of *CsCLV3*. Auxin also influences carpel number through interaction between auxin response factor CsARF14 and CsWUS [60]. 

Within each carpel, the ovules are arranged in a linear array along the longitudinal axis (Figure 2). Ovule number, which determines maximum seed number, is positively correlated with ovary and fruit length. QTL for ovule number co-localized with QTL for fruit length on chromosomes 1 and 6 [6]. The location of the ovule number on chromosome 6 in cucumber falls in a region of synteny with a QTL for ovule number on chromosome 8 in melon [61], suggesting possible shared genetic basis, although, the underlying gene(s) remain to be determined. Several lines of evidence indicate a key role for auxin in ovule development. Mutation in the cucumber ortholog of *PINOID* (*CsPID*), a protein kinase that mediates polar auxin transport, has widespread effects on cucumber plant development [62]. The *CsPID* mutant, which was originally named *rl* for round leaf, also produces fruit with rounder apices that are devoid of ovules. The *CsPID* mutant plants exhibit greatly decreased auxin levels in developing ovaries, and decreased expression of key ovule morphogenesis genes including *CsWOX1, CsWUS,* and *CsSPL (SPOROCYTELESS)* [62,63]. Similarly, CsSPL stimulates expression of auxin response factor *CsARF3* and is positively regulated by CsARF13 [63]. Mutation of the mango fruit (*mf*) gene encoding CsWOX1, which directly interacts with CsSPL, also affects auxin distribution; the recessive *mf* gene causes a poorly developed seed cavity, poorly-defined separation of the three carpels, and reduced growth at the blossom end of the fruit leading to a tapered fruit shape [64]. 

The most frequently used measure of fruit shape, the fruit shape index (FSI), is the length/diameter (L/D) ratio, a parameter that is also used for market class designations and grading standards. Fruit length, which has a high heritability, appears to be the primary driver of fruit shape and is highly correlated with fruit shape index throughout fruit growth [28,65,66,67]. Within cucumber germplasms, there is extreme variation in length. Cucumber fruit also vary in diameter and can range from highly elongated to nearly round (Figure 1). While auxin content was positively correlated with fruit length, it was negatively correlated with fruit diameter [5]. Fruit diameter also appears to be more subject to environmental variation and is less well correlated with FSI than fruit length [6,26,28,67]. Several studies have found an absence of correlation between fruit length and diameter among segregating progeny, indicating potentially independently acting factors for longitudinal vs. radial growth [6,68,69]. Numerous studies have identified QTL associated with ovary and fruit shape index (L/D), resulting in one or two consensus QTL on each chromosome (reviewed in [28,30]). While many QTL are shared for length and diameter, distinct QTL affecting either length or diameter also have been identified. 

Cell count analysis indicated that differences in orientation of cell division pre-anthesis was a key factor influencing ovary shape, and ultimately fruit shape [4]. Not surprisingly both fruit length and diameter were strongly correlated with cell number in longitudinal or cross section, respectively [4,5]. QTL analyses, candidate gene analysis, and narrowing of a key fruit shape locus through the use of chromosome segment substitution lines indicated that a homolog of the tomato fruit shape gene *SUN* is a prime candidate for the gene underlying *FS/FSI1.2* [70,71]. *SUN* in tomato encodes an IQ67 domain-containing protein that influences the orientation of cell division pre- and post-anthesis [72]. A deletion in the first exon of *CsSUN* was associated with round fruited cucumbers [70]. *FS1.2* is also very closely linked with the *M* locus (0.2 Mb) [28], possibly contributing to the frequently observed association between flower sex and fruit shape. A second consensus locus, *FSI2.1*, has been predicted to encode a homolog of the OVATE-interacting factor, *TRM5,* consistent with a model shared across several species of interaction between TRM and OVATE FAMILY PROTEINS (OFP) to regulate plant organ shape, including fruit shape [70,73]. Post-pollination, the increase in length and diameter both follow a typical sigmoidal growth curve; however, relative timing of peak growth can vary with length vs. diameter, influencing FSI at different stages of fruit development (e.g., [4,28,74]). Differential extent and orientation of cellular expansion influencing cell size and shape also can contribute to differences in cucumber fruit shape [4]. 

Another variable feature is the presence of a stalk or neck, located at the peduncle end of the fruit and superior to the seed cavity (Figure 2) [75]. The neck accumulates high concentrations of the trisaccharide raffinose, rather than monosaccharides, as occurs in the seeded portion of the fruit, indicating tissue-specific sugar metabolism and transport [76]. A pronounced neck is characteristic of many European greenhouse cucumber cultivars and also can be observed in Chinese Long cucumbers (Figure 1c). During the first few days following pollination, the neck undergoes rapid cell expansion rather than cell division [76]. A candidate gene for neck length (*CsFnl7.1*) encoding a late embryogenesis abundant protein was identified via fine mapping, gene expression analysis, and overexpression in transgenic cucumbers [77]. CsFnl7.1 was found to modulate cell expansion, possibly by interaction with a dynamin-related protein and a germin-like protein. A second gene, *HECATE1* (*CsHEC1*), also has been demonstrated to influence neck length; increased *CsHEC1* expression is associated with increased neck length [78]. CsHEC1 binds to the promoter of the auxin biosynthesis gene, *YUCCA4* (*CsYUC4*), leading to enhanced expression of *CsYUC4* and increased neck growth. The auxin-promoting effect of CsHEC1, however, was shown to be modulated by direct interaction with CsOVATE, which in turn reduced the ability of HEC1 to stimulate expression of *CsYUC4*. 

Commercially grown cucumbers are typically straight, i.e., oblong or cylindrical, cucumbers; however, accessions can exhibit one or more curves leading to extremes of circular or S-shaped fruit, especially in highly elongated fruit (Figure 1). Fruit curvature is a negative trait for cucumbers, lowering its marketability. Analysis of the convex and concave regions of cucumber fruit during the phase of active cell division and early expansion, 2–6 days post anthesis, showed asymmetric distribution of auxin and differential expression of auxin-related genes, including the auxin (*YUCCA*) biosynthesis gene, *CsYUC10b* [79]. Treatment with auxin or overexpression of *CsYUC10b* reduced the curvature angle of developing fruits, providing another example of the important role auxin plays in multiple aspects of cucumber fruit growth and development. Other features than can reduce commercial value are sharp fruit apex angle and fused fruit. QTL analysis of fruit apex angle identified four major effect QTL, two on chromosome 4 and two on chromosome 6 [80]. Fused fruit, *twin fused fruit* (*tf*), is the result of two pistillate flowers with partially joined ovaries developing from a single peduncle [81]. The recessive *tf* trait was only observed in gynoecious plants, indicating epistasy between *F* and *t*

## 3. Fruit Surface Features

The surface of cucumber fruits can vary extensively with respect to waxes, number, size, and shape of warts (also called tubercules), density and types of trichomes, and presence of ‘bloom’, a fine white powder that can give the fruit a dull appearance (Figure 1 and Figure 2). The cuticle layer and associated waxes which limit water loss can influence fruit appearance, shipping and handling procedures, and produce shelf life. The number and size of warts can be a defining feature of market type. While Western fresh market cucumbers are generally smooth at time of harvest, Western pickling cucumbers typically have a surface with infrequent, rounded warts, and north Chinese type cucumbers have extensive, large warts covering the fruit surface. As presence of warts and spines can impede packing and handling of fruit, fruits with a smoother surface are increasingly favored for commercial production. 

### 3.1. Trichomes

Cucumber fruit are often marked by the presence of warts comprised of tubercules (raised fruit surface) topped with spines (Figure 2). The spines are large, multicellular, non-branched, non-glandular trichomes (type II in tomato trichome nomenclature), composed of an enlarged base and pointed stalk [82]. Although spines are most frequently located on tubercules, the two traits can be disassociated, i.e., spines can be present without tubercules [83]. They also are typically absent from the neck portion of fruits (Figure 1 and Figure 2). The round base of the trichome, which is clearly visible on the ovary at anthesis, contains hundreds of spherical cells and is especially large relative to other species and other parts of the cucumber plant [84,85]. Height and sharpness of the spine is conferred by the stalk, which is formed from several, progressively narrower, cylindrical cells situated one on top of the other [82]. The spines are initiated during early ovary development and are highly visible at anthesis where differences in presence/absence, density, and color are readily observed (Figure 1a). Post-pollination the spines follow a developmental progression, frequently aborting as fruits approach full size. In pickling cucumbers, spine color changed with development from translucent or light green at anthesis, to yellow, to white at the time of abscission (12–16 dpp) [11]. 

The initiation and development of cucumber fruit spines has been the subject of multiple investigations in recent years leading to identification of numerous genes involved in spine establishment and structure. Spine density results from location of spine initiation. GWAS of diverse cucumber germplasms identified nine loci associated with naturally occurring variation in spine density [83]. A trait conferring numerous spines (*ns*), was initially mapped to the center of chromosome 2 [86]. The non-functional version of NS, leading to increased spine density, was found exclusively in Eurasian cucumber accessions [83]. A combination of fine mapping, functional analysis via overexpression and CRISPR knock out, and fruit peel specific expression indicated that the *ns* trait resulted from mutation of an AUX1/LAX-type auxin transporter gene, *CsAUX1* [83,87]. Fruit peels of the *ns* mutants and *ns* knock outs exhibited reduced auxin levels and decreased expression of Aux/IAA transcriptional repressors, indicating a central role for auxin in initiation of cucumber fruit spines. Other loci associated with naturally occurring variation in spine number include QTL on chromosome 6, *fsd6.1* and *fsd6.2* [88]. *fsd6.2*, which originated in China, promotes ultra-high spine density when in combination with *fsd6.1*. Fine mapping of *fsd6.2* identified a homolog of the tomato HD-ZIP IV factor *Wooly*, essential for multicellular trichome formation in tomato [89], as the candidate gene for *fsd6.2* [named *Csgl3 (glabrous3)* in cucumber] [88]. 

In addition to naturally occurring variation for spine density, numerous mutants that influence both spine initiation and development have been identified, providing evidence of the complexity of spine formation. As might be expected, many are homologs of transcripition factor genes regulating trichome formation in Arabidopsis or tomato. Additional allelic variants of the above-mentioned glabrous *Csgl3* HD-ZIP class IV factor gene have been identified [e.g., *csgl3, tril (trichomeless)*] [90,91,92,93,94,95] and *CsTTG1*, a homolog of Arabidopsis *TRANSPARENT TESTA GLABRA1 (TTG1),* encodes a WD-repeat homolog protein [84]. *CsTRY* and *CsMYB6*, encode homologs of Arabidospsis negative transcription factors, *TRIPTYCHON*, and a MIXTA-like MYB transcription factor, *AtMYB106*, respectively [96]. Studies of cucumber *glabrous-1 (csgl1*) [alternate names: *micro-trichome* (*mict*) and *tiny branched hair* (*tbh*)] found that all were mutations of the same gene encoding a member of the Class I homeodomain-leucine zipper (HD-ZIP) transcription factor family [82,90,97,98]. The TBH protein was found to bind promoters of ACS genes increasing ethylene and female flower production, indicating a possible role for ethylene in trichome formation [99]. In Arabidopsis, HD-ZIP class I factors play critical roles in plant development and are frequently involved in abiotic stress responses including abscisic acid, ethylene, and jasmonic acid signaling [100,101]. In addition, several NAC family transcription factors and associated microRNAs have been implicated to play a role in trichome initiation [102]. Despite extensive conservation across species for key genes associated with trichome development, several studies also have identified unique interactions among the different factors governing spine initiation in cucumber fruit. 

Cucumber fruit spines exhibit morphological variability with respect to size, shape, and pigmentation. Notably, several of the genes implicated in trichome initiation also appear to influence subsequent trichome development. A primary determinant of spine size is size of the base, as indicated by genes such as *spine size* (SS) and *spine base size 1* (*SBS1*) [103,104]. *SBS1* encodes a C2H2 zinc-finger transcription factor that was shown to form a trimeric complex with the spine density factors, *CsTTG1* and *CsGL1* [104]. Overexpression of *CsTTG1* also causes larger and longer spines due to an increase in both cell number and cell size [105]. Unlike *Csgl3* mutants which lack trichomes, the *Csgl1* (*mict, tbh*) alleles produce equivalent but very small numbers of aberrantly shaped trichomes [82,97,98]. The mutation *nps* (no pyramid-shaped head), which caused smaller and more densely packed trichomes, was found to be an allele of *mict* (*Mict-L130F*) [106]. Gibberellins also have been implicated to play a role in trichome development, as overexpression of CsGA20ox1 caused shorter fruit spines [9]. 

Spine colors range from clear to heavily pigmented due to accumulation of anthocyanins. Black or brown spines, which are dominant to clear (white) spines, have long been associated with the *B* locus, although other genes also have been reported to influence spine color. The *B* gene, *CsMYB60,* encodes an R2R3-MYB type regulatory protein [107,108,109]. Overexpression and induced expression of the anthocyanin biosynthetic pathway increased production of flavanols and proanthocyanadins. Lack of pigmentation resulting from a *MULE*-like transposon insertion was found extensively in cucumbers from East Asia and Australia [109]. 

The second predominant type of trichomes are glandular ‘bloom trichomes’. These trichomes secrete silica oxide, causing the dusty appearing ‘bloom’ on the fruit surface [110]. Like spines, the presence of bloom and bloom trichomes also changes with fruit growth [97]. In pickling cucumber, the bloom progressively disappeared from the stem to blossom end over the first two weeks of development [11]. CsTTG1 acting in concert with CsGL1(Mict) has been implicated in formation of both types of trichomes [84,97]. Overexpression increased density of both spines and bloom trichomes. However, it appears that the two types of trichomes are also somewhat independently regulated, as plants with the *Csgl1* mutation that eliminate spine trichomes still initiate bloom trichomes, but they do not complete development [97]. 

### 3.2. Tubercules

The tubercules are a raised structure on the fruit surface formed by several additional layers of cells [111]. Formation of tubercules and spines are closely related. Spines are typically formed on top of tubercules, and glabrous *CsGL1* and *CsGL3* mutants fail to produce tubercules, indicating that presence of spines is required for presence of tubercules [82,90,111]. Consistent with the epistasis of spine formation to tubercules, tubercule formation is regulated by a dominant gene, *CsTu*, encoding a C2H2 zinc finger transcription factor that is expressed specifically in the fruit spine cells during development of tubercules [111]. *CsTu* is not expressed in *CsGL1* mutants lacking trichomes. The MIXTA-LIKE transcription factor CsMYB6 also influences both spine and tubercule formation by interacting with CsTTG and CsTu, respectively [112].

During the first few days post pollination, warts rapidly increase in size, becoming increasingly prominent (Figure 3) [11,111]. Cytokinins and auxins have been implicated to promote wart development. *CsTu* and a second wart-associated gene, the basic Helix-Loop-Helix (bHLH) gene *HECATE2* (*CsHEC2*), promote cytokinin production [111,113]. CsHEC2 binds to the promoter of the cytokinin biosynthesis gene, CTK hydroylase-like1 (*CsCHL1*), and also interacts with CsGL3 and CsTu, further increasing CsCHL1 expression [113]. CsTu also binds to the promoter and promotes expression of the tubercule size gene, *Tubercule size 1* (*CsTS1*) [114]. Expression levels of *CsTS1* conferred by allelic variation in the promoter region were directly correlated with wart size in a range of cucumber accessions. Increased expression of *CsTS1*, which encodes an oleosin protein, also increased auxin levels, promoting cell size of the tubucules. For many market types, as the fruit continues to expand, warts progressively flatten (Figure 3); in other market types, warts may remain pronounced throughout the exponential growth period (Figure 1b).

### 3.3. Cuticle and Wax

The cuticle is a hydrophobic layer covering the aerial surfaces of plants that functions to limit water loss, provide mechanical support for fruit growth and development, and protect against environmental stresses, such as pathogens, insects, UV radiation, and drought. The cuticle is composed of a matrix of polymerized cutin monomers (epoxy-hydrolxylated C16 and C18 fatty acids) and waxes [115,116]. Additional secondary metabolites such phenylpropanoids, triterpenoids, flavonoids, and sterols may also be found in the cuticle. Cuticle and wax substrates are produced and secreted by epidermal cells of fruit peel, where deposition is developmentally programmed during fruit growth and is largely complete by the end of late exponential growth [115,116]. The composition and thickness of cucumber fruit cuticles and expression of cuticle-associated genes vary by developmental age, variety, and environmental conditions, with peak deposition corresponding to the exponential growth phase [117,118]. 

As the cuticle is the most exterior part of cucumber fruit, it can influence surface appearance of fruit through its structure and composition and type of epicuticular waxes, which can influence glossiness, uniformity of appearance, and susceptibility to cracking and damage [115,116,119]. Several genes associated with cutin and wax biosynthesis have been identified to influence natural variation in cucumber fruit glossiness. A QTL for fruit skin gloss in cucumber (*CsFSG1*) was mapped to a 0.1 Mb region of chromosome 3 using bulk segregant and genome-wide association studies [120]. Of 11 candidate genes in the region, only the cytochrome P450 family gene *CsCYP86B1* showed increased expression in early fruit development between high-gloss and low-gloss near isogenic lines. Members of the CYP86 subfamily encode fatty acid ω-hydroxylases that are involved in cutin synthesis in Arabidopsis [121] and glossiness of fruit in tomato is highly impacted by cutin synthesis, where cutin-deficient mutant lines show increased glossiness [122].

Another study comparing cucumber lines with glossy and waxy phenotypes found the greatest difference in gene expression in the preferentially fruit epidermal-expressed gene, *CsECERIFERUM 1 (CsCER1)* [123]. Similar to Arabidopsis CER1, CsCER1 contains three His-rich motifs constituting the fatty acid hydroxylase domain necessary for sterol desaturase activity, and in the C-terminal region, there is a functionally uncharacterized WAX2 domain needed for alkane biosynthesis. Electron microscopy analysis of transgenic cucumber overexpression and RNA interference (RNAi) lines displayed more wax crystals in *CsCER1* overexpression lines and a deficiency of epicuticular wax crystals in *CsCER1*-RNAi lines. Gas chromatograph-mass spectrometry (GC-MS) analysis of cuticle composition showed that the alteration in wax load in the transgenic *CsCER1*-RNAi lines resulted from decreased very-long-chain (VLC) alkane content. A second gene involved in cucumber wax biosynthesis via alkane production, *ECERIFERUM 3 (CsWAX2)*, appears to impact both wax and cutin biosynthesis [123], and a third gene, *CsCER4,* which is involved in wax synthesis through primary alcohol formation, is more highly expressed in glossy cucumber fruit during early fruit development [124].

Natural variation in cucumber fruit surface properties is also associated with transcriptional regulation of cuticle development. QTL and transcriptomic analyses indicated the transcription factor *CsSHINE1/WAXINDUCER1* (*CsSHN1/WIN1*) influences cuticle thickness, depth of cuticular intercalations between epidermal cells, and epidermal cell shape in a comparison of North American pickling and Asian fresh market varieties with thick and thin cuticles, respectively [118]. *SHN1/WIN1* is part of the apetala2/ethylene-responsive element binding protein (AP2/EREBP) family of transcription factors and has been shown in other systems to influence cuticle deposition and epidermal cell shape [125,126]. *CsSHN1* was preferentially expressed in cucumber peel tissue, where peak expression occurred during the exponential growth phase of fruit development, coinciding with peak cuticle deposition. Two SNPs were identified in *CsSHN1* in the Asian fresh market variety Chinese Long ‘9930′ [118,127]. One SNP confers an amino acid substitution for methionine in the first exon, which results in loss of the regular start codon as well as the AP2 domain necessary for proper function. When examining the allelic diversity among 160 cucumber accessions, this SNP was found to be a rare allele [127]. The other SNP located in exon 2 results in an amino acid substitution of proline by arginine in a highly conserved CMV-1 domain. This variant is found predominantly in East Asian accessions [118].

### 3.4. Netting

A related trait, though infrequently observed in commercial cucumber, is netting of the fruit surface. Cracking of the fruit skin can occur when the fruit skin is incapable of withstanding the internal pressure that is created as fruits expand in volume [128]. If the fissures extend to cellular layers below the cuticle, a rough, corky matrix of wound-periderm tissue composed of specialized cells that have been suberized and/or lignified forms below the fissures. These fissures can interconnect giving a netting pattern, also known as russeting or skin reticulation, as is typical of many melon (*Cucumis melo*) cultivars [129]. Sikkim cucumbers (*Cucumis sativus* var. *sikkimensis*) have a wound-periderm resulting from extensive skin cracking throughout development. Microscopy and GC-MS analyses revealed the presence of phenolic-rich suberin in the fruit skin of mature Sikkim cucumbers that was not present in skin of mature common cucumbers [130]. QTL analysis found that the *Rs* (russet skin) locus controlled netting in Sikkim cucumber and could be mapped to a 736 kb region on chromosome 1 [131]. *CsSHN1/WIN1* was identified as the candidate gene for the *Rs* locus through map-based cloning [127]. Copy number variations of *CsSHN1/WIN1* contributed to the degree of netting observed in different cucumber lines with two functional copies in a Sikkim cucumber line with heavy netting, whereas a pickling line of cucumber that had a single functional copy of *CsSHN1/WIN1* had a light skin netting phenotype [127]. An additional dominant locus identified within *C. sativus* germplasm conferring *Heavy netting* (*H*) has been mapped to chromosome 5 [69]. While a candidate gene has not been identified, QTL analysis and linkage mapping with new markers narrowed the region to 271 kb [131].

## 4. Fruit Color

### 4.1. Skin Color

The skin of mature cucumber fruit can exhibit a range of colors, e.g., white–green–yellow–orange–brown (Figure 1d). However, skin color of harvest-stage, immature cucumber fruit, like other immature fruit has a narrower spectrum, ranging from white to light green to dark green. It has been suggested that variation in immature fruit skin color may produce desirable novelty for the market. As might be expected, a greater number of chloroplasts and higher levels of chlorophyll a, chlorophyll b, and carotenoids have been observed in fruit with dark skin compared to that with light green skin, and metabolic analyses suggest that darker green fruit peels may also have higher content of flavonoids and anthocyanins as well as greater antioxidant activities [132,133]. A recessive white immature fruit trait has been associated with QTL on chromosomes 3 and 5 [134]. Fruit with lighter colors also have been produced via mutagenesis, influencing genes such as a Ycf54-like protein implicated in the cyclase step of chlorophyll biosynthesis [135]; a gene homologous to Arabidopsis ARC5 important for chloroplast division [136]; the transcription factor, MYB36 reducing chlorophyll content and causing a yellow green peel [137]; and a GATA transcription family member that has been implicated in other systems to regulate chlorophyll synthesis [138]. Mutations resulting in white fruit with fewer chloroplasts and reduced chlorophyll content include a premature stop in the *w* gene encoding a putative two-component response regulator (RR)-like protein, APRR2 [139,140,141]. Fruit with lighter color skin also can be observed in cucumber diversity panels and are likely the result of naturally occurring mutations in chlorophyll-related genes. 

### 4.2. Flesh Color

A narrower range of color is observed in the fruit mesocarp and endocarp. Cucumber fruit flesh is typically white; however, variants with yellow, orange, and green flesh are observed (Figure 1e). Yellow and orange flesh result from carotenoid accumulation [142,143], including beta-carotene. As beta-carotene is a precursor of vitamin A, orange flesh is of interest to improve nutritional content. The endocarp of Xishuangbanna cucumber fruit is noted for accumulation of high levels of beta-carotene in the endocarp attributed to natural variation in a beta-carotene hydroxylase gene [142]. A second gene associated with orange flesh, *CsOr* (orange), encodes a DnaJ cysteine-rich zinc finger domain chaperone protein associated with induction of beta-carotene accumulation [143]. *Or* gene homologs previously have been associated with orange phenotypes in a variety of plant species (cauliflower, potato, sweet potato), including melon [144]. Green flesh in cucumber, like other parts of the plants, results from accumulation of chlorophyll. Major-effect QTL influencing green flesh color were identified on chromosomes 3 and 5, although the underlying genes remain to be determined [145]. 

## 5. Internal Features

The interior of the cucumber fruit consists of mesocarp, the edible fleshy part; endocarp, a soft, juicy pulp surrounding the seeds; and seeds (Figure 2). The fruit develop from 3–5 carpels that fuse together during double fertilization and form a transmitting tract, extending from the stigma to the base of the ovary, through which pollen tubes grow to reach the ovules. As the carpels compress together and fuse, the cell layers at the ventral side become irregular and unrecognizable [146]. The center of the fused carpels becomes the seed cavity and the enlarged mesocarp becomes the fruit flesh. The proportion and characteristics of these structures define edibility and market values of different cucumber varieties. For cucumber fruit, smaller seed cavity size is preferable, which is usually accompanied by thicker flesh.

### 5.1. Flesh Thickness

Flesh thickness is an important fruit quality trait that has been selected during 3000 years of domestication [142]. During the expansion phase of fruit growth, mesocarp cells become highly vacuolated and rapidly increase in size leading to increased flesh thickness [147]. A flesh thickness QTL has been identified in a 0.19 Mb region on chromosome 2 (*fft2.1*) in a segregating F_2_ population [148]. Of 20 genes found in this region, *Csa2M058670.1* was highly expressed in the thick fruit flesh parent but not the thin flesh variety, and sequence alignment showed a 4 bp deletion in the promoter region associated with the thin flesh phenotype [148]. *Csa2M058670.1* encodes a SET domain protein-lysine methyltransferase (PKMT) protein, a member of the SET domain gene family, which in Arabidopsis has been shown to be involved in cell division and differentiation [149]. A transcriptomic study on the same two varieties found that many auxin- and cytokinin-related genes were more highly expressed in the mesocarp of the thick flesh variety at 0–9 days after anthesis (DAA), suggesting that auxin and cytokinin may contribute to mesocarp development [150]. A recent study of selective signatures in different cucumber populations showed evidence of specific selection at the *Csa2M058670.1* locus in the Xishuangbanna population, where thinner fruit flesh is more prominent [151]. Additional QTL influencing flesh thickness were found on chromosomes 3 and 5 (*fth3.1* and *fth5.1*) from two Sikkim-type cucumber (*C. sativus* var. *sikkimensis*) segregating populations [131]. While *fth3.1* increases flesh thickness in WI7088D, *fth5.1* does the opposite. These findings imply that multiple population-specific QTL may control the trait. Flesh thickness QTL also co-localize with QTL for other fruit size and shape traits such as fruit length, diameter, and fruit shape index [30]. 

### 5.2. Seed Cavity and Hollowness

The development of seed cavity size is highly correlated to fruit diameter growth, growing exponentially from 0–12 DAA, then slowly increasing until maturity [5]. The first QTL for seed cavity size was reported in 1995 [152]. Subsequently different QTL were identified from different seasons and locations [30,71,153], suggesting that seed cavity size is highly affected by both genotype and environment. Cucumber fruit also can exhibit a hollow region in the interior of the fruit (Figure 1e). Hollowness, which results from a failure of the carpels to fully fuse after fertilization, reduces fruit quality [146]. For pickling cucumber, hollowness is especially undesirable since it can cause bloating during brining and reduce economic value [154]. In Arabidopsis, *SPATULA* (*SPT*) and *ALCATRAZ* (*ALC*) control transmitting tract differentiation. The double knockout mutant of these genes in cucumber (*Csspt Csalt*) causes a hollow center [155]. Lignin was found to accumulate between the cell layers of different carpels in the *Csspt Csalt* mutant, resulting in carpel separation and hollowness formation. In addition, *Csspt Csalt* double mutants also lost female fertility caused by stigma and style deformation, and no seeds were produced. However, hollowness per se is not an indicator of plant fertility as it is commonly observed in cucumber germplasms, such as Sikkim-type cucumber, which produce healthy seeds despite large hollows in mature fruits [155]. An additional gene related to seed cavity and hollowness formation, *CsALMT2*, encodes an aluminum-activated malate transporter (ALMT); *CsALMT2* was significantly downregulated in a breeding line that stably formed hollows [146]. *CsALMT2* was highly expressed in fruits and roots and located on the plasma membrane in subcellular localization analysis of tobacco leaves [146]. *CsALMT2* may regulate the distribution of organic acids in cucumbers as its watermelon homolog regulates malic acid accumulation in vacuoles in pulp tissue during fruit development [156]. 

## 6. Conclusions

Numerous studies in recent years have added greatly to our understanding of cucumber fruit development and have identified a variety of genetic factors leading to extensive morphological diversity. At the earliest stages of development, genes influencing floral organ establishment, including sex expression and carpel number, set the stage for fruit shape [50,58,60,61,62,63,64]. Subsequently, critical components influencing fruit size and shape include timing and level of expression of genes involved in cell division and cell cycle regulation during ovary development and early fruit set [3,12,13,15,16,148]. Not surprisingly, many of the genes affecting cell division, both pre- and post-pollination, have been shown to be associated with modified expression of genes involved in hormone biosynthesis, transport, degradation or inactivation, which in turn, influence extent and orientation of cell division (e.g., [15,16,21,23,79,150]). Genes influencing cell expansion via sugar transport and phloem unloading also can contribute to variation in size, shape, and internal fruit structure [18,19,146]. Homologs of known fruit size and shape genes identified in other species have been found within consensus cucumber fruit size and shape QTL and may also contribute to diversity in cucumber fruit morphology [28,30]. However, in most cases, their specific involvement in cucumber fruit development remains to be verified. Our understanding of genetic control of morphological features such as spines, warts, color, and waxes also has greatly increased in recent years. The presence and density of trichomes and warts has been associated with homologs of numerous transcription factors regulating trichome development in other species as well as genes influencing auxin and cytokinin levels and transport [87,88,90,96,97,98,111,113,114]. Variation in traits such as cuticle thickness and composition and skin and flesh color has been found to be mediated by structural genes encoding biosynthetic enzymes for cutin, wax, chlorophyll, and beta-carotene synthesis, as well as transcription factors regulating cuticle deposition and pigment biosynthesis [118,120,124,127,135,136,137,138,139,140,141,143]. Collectively, these findings demonstrate the complex interplay between structural genes, transcription factors, and hormone signaling leading to morphological diversity in cucumber. Looking ahead, we can only expect that with expanding genetic, genomic, and bioinformatic tools, our understanding and ability to manipulate these traits will continue to increase. Identification of genetic factors controlling these traits will facilitate breeding for improved characteristics to increase productivity, improve shipping, handling, and storage properties, and enhance consumer-desired qualities.

## Data Availability

Not available.
